# Combinational analysis of linkage and exome sequencing identifies the causative mutation in a Chinese family with congenital cataract

**DOI:** 10.1186/1471-2350-14-107

**Published:** 2013-10-08

**Authors:** Xueyuan Jia, Feng Zhang, Jing Bai, Linghan Gao, Xuelong Zhang, Haiming Sun, Donglin Sun, Rongwei Guan, Wenjing Sun, Lidan Xu, Zhichao Yue, Yang Yu, Songbin Fu

**Affiliations:** 1 Laboratory of Medical Genetics, Harbin Medical University, Harbin 150081, China; 2 Ministry of Education Key Laboratory of Contemporary Anthropology and State Key Laboratory of Genetic Engineering, School of Life Sciences, Fudan University, Shanghai 200433, China; 3 Key Laboratory of Medical Genetics (Harbin Medical University), Heilongjiang Higher Education Institutions, Harbin, China

**Keywords:** Autosomal dominant congenital cataract, Whole exome sequencing, Linkage analysis, *CRYGD*, Coralliform cataract

## Abstract

**Background:**

Congenital cataract is a Mendelian disorder that frequently causes blindness in infants. To date, various cataract-associated loci have been mapped; more than 30 genes have been identified by linkage analysis. However, the pathogenic loci in some affected families are still unknown, and new research strategies are needed. In this study, we used linkage-exome combinational analysis to further investigate the pedigree of a four-generation Chinese family with autosomal dominant coralliform cataract.

**Methods:**

We combined whole exome sequencing and linkage analysis to identify the causative mutation. The exome capture and next-generation sequencing were used to sequence the protein-coding regions in the genome of the proband to identify rare mutations, which were further screened for candidate mutations in linkage regions. Candidate mutations were independently verified for co-segregation in the whole pedigree using Sanger sequencing.

**Results:**

We identified a C to A transversion at nucleotide position c.70 in exon 2 of *CRYGD*, a cataract-associated gene. This mutation resulted in a threonine substitution for proline at amino acid residue 24.

**Conclusions:**

We identified a missense P24T mutation in *CRYGD* that was responsible for coralliform cataract in our studied family. Our findings suggest that the combination of exome sequencing and linkage analysis is a powerful tool for identifying Mendelian disease mutations that might be missed by the classic linkage analysis strategy.

## Background

Congenital cataract is a Mendelian disorder resulting in blindness during infancy or early childhood. Non-syndromic congenital cataracts have an estimated frequency of 1 to 15 per 10,000 live births throughout the world [1-3]. Congenital cataract is primarily autosomal dominant, although autosomal recessive and X-linked inheritances have also been reported [4]. To date, various cataract-associated loci have been mapped, in which more than 30 genes were identified by linkage analysis. Most cataract-associated genes are crystallin genes, such as alpha crystallins (*CRYAA* and *CRYAB*), beta crystallins (*CRYBB1*, *CRYBB2*, *CRYBB3*, *CRYBA1*, *CRYBA3*, and *CRYBA4*), and gamma crystallins (*CRYGA*, *CRYGC*, *CRYGD*, and *CRYGS*). Approximately 25% of affected families have defects in membrane transport genes, including major intrinsic protein of lens fiber (*MIP*), gap junction proteins (*GJA3* and *GJA8*), transmembrane protein 114 (*TMEM114*), and lens intrinsic membrane protein 2 (*LIM2*). The remaining known mutations are found in genes encoding cytoskeletal proteins, growth and transcription factors, v-maf musculoaponeurotic fibrosarcoma oncogene homolog, heat shock transcription factor, and others as outline in Additional file 1: Table S1.

Linkage analysis is a classic strategy for mapping disease-associated loci in Mendelian inheritance pedigrees. This method requires large families, commonly a multi-generation pedigree with at least 6 to 12 affected individuals, to obtain high reliability and statistical significance. However, significant linkage remains hard to establish despite large sample sizes, particularly due to a low density of microsatellite markers, misclassification of patients, low heterogeneity, low disease penetrance, or clinically identical phenocopies [5]. Furthermore, although significant linkage may be obtained, it is still difficult to further identify causal mutations in a large genomic interval including dozens of genes [6,7]. Next-generation sequencing (NGS) technology provides new avenues for uncovering genetic causes of human diseases. Although whole genome sequencing is becoming more practical due to its falling cost and increased throughput, it still remains expensive for most applications. Whole exome sequencing is an economical method compared to whole genome sequencing. Recent studies showed that the human genome contains about 180,000 exons, accounting for about 1% of the total genome [8]. Thus, whole exome sequencing is especially promising for research on monogenic disorders [9,10] since most of these disorders are caused by exonic mutations or splice-site mutations.

In a previous study, we presented evidence to suggest some candidate linkage regions in a four-generation Chinese family with autosomal dominant coralliform cataract, but the causal mutation was not identified [11]. In the current study, we have further investigated the same pedigree using whole exome sequencing and linkage combinational analysis to identify the causal mutation.

## Methods

### Clinical ascertainment and DNA sampling

**Figure 1:**
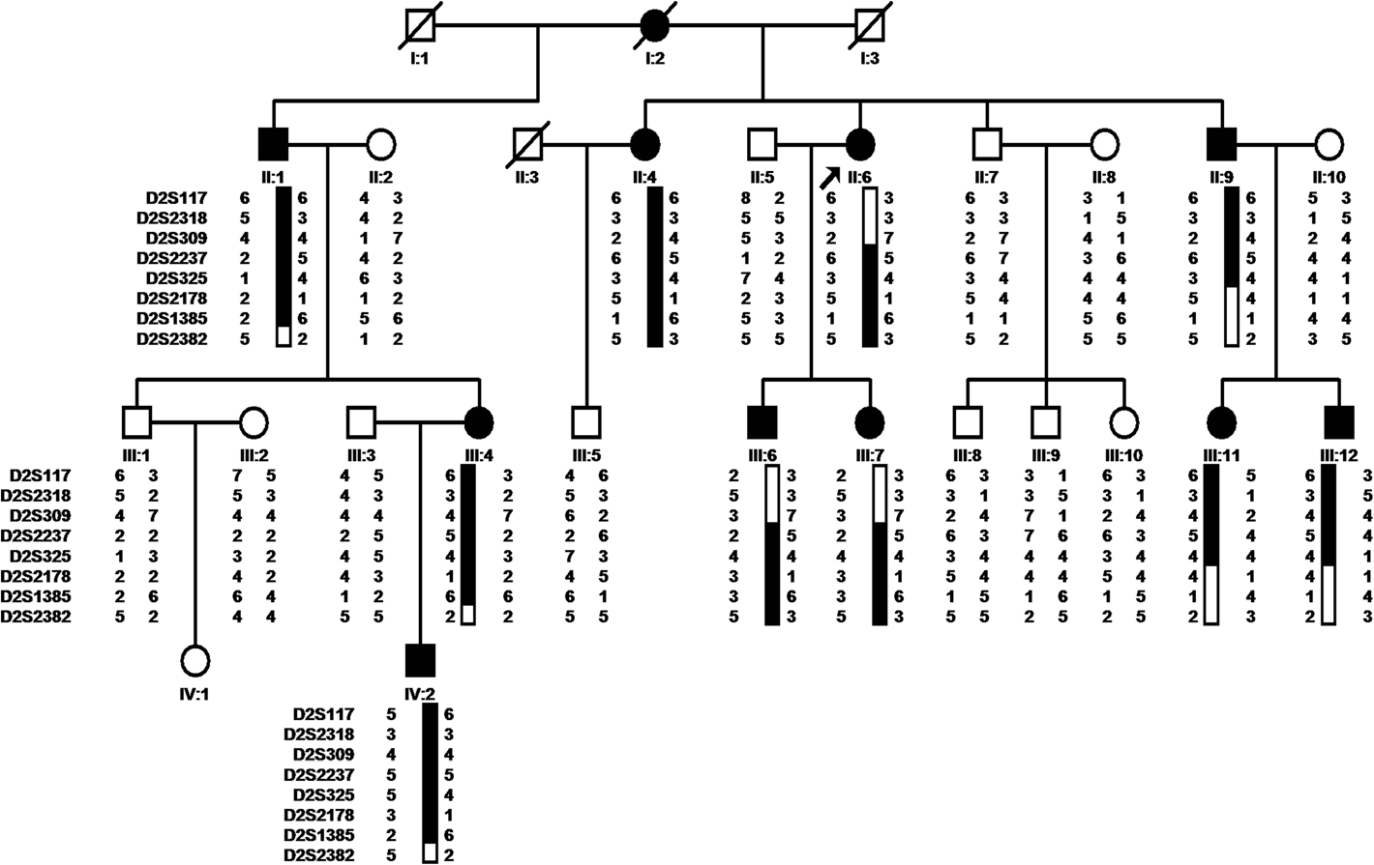
**Pedigree of a four-generation Chinese family with autosomal dominant coralliform cataract and the linked haplotypes.** The black arrow indicates the proband. Black symbols and bars denote affected status.

A total of 19 family members, including 9 affected and 10 unaffected individuals, were recruited for a previous study by Gao et al. in 2005 [11]. Three additional individuals (affected III:11, unaffected III:5 and III:8) were newly recruited for this study (Figure 1). All members of this family underwent an examination that included photography and slit-lamp microscopy of the lens. The research project was approved by the Ethics Committee of Harbin Medical University. All samples were collected with informed consent from the participants. Written informed consent was obtained from the parents of each child. All experiments carried out with human subjects were in compliance with the Helsinki Declaration.

DNA samples were extracted from peripheral blood leukocytes using the QIAamp Blood Mini DNA kit (Qiagen, Santa Clara, CA, USA). Before analysis of the samples, DNA aliquots were re-precipitated to remove proteins and fragments.

### Exome capture and next-generation sequencing

A whole exome–enriched library was prepared from 3 μg of genomic DNA from the proband (II:6) using Agilent’s SureSelect Human All Exon 50 Mb solution-based capture reagent. Exome capture was performed according to the manufacturer’s protocol (Agilent, USA). The captured DNA was then sequenced using the Illumina HiSeq2000 platform. Raw image files were processed by Illumina Basecaller Software 1.7 (San Diego, CA, USA) for base-calling with default parameters.

### Short-read alignment, mapping statistics, and variant annotation

The obtained sequence reads were aligned to the human genome (hg19) using the SOAP2 [12] and BWA [13] tools for single nucleotide polymorphism (SNP) and insertion/deletion (indel), respectively. The percentages of read alignment to both the reference genome and the targeted exome were calculated using Perl scripts. Similarly, Perl scripts were used for the detection of mismatch frequencies and error positions. SNP calling was done with SOAPsnp [14], and indels were identified through the alignment result with GATK [12]. Detailed annotation information was obtained from dbSNP, CCDS, UCSC Genome Browser, Ensembl, and Encode databases. Using these annotations, we screened the novel and likely deleterious variants for further study.

### PCR and Sanger sequencing

Specific primers were designed for the target region, and the PCR products were sequenced on an ABI 3730 DNA analyzer following standard procedures (Life Technologies, USA). The sequence reads were analyzed using the Sequencher software package (GeneCodes Inc, USA). The sequencing traces were visually inspected in Finch TV v1.4 (Geospiza Inc, USA).

### Linkage and haplotype analysis

Microsatellite markers were selected based on ABI PRISM Linkage Mapping Set (version 2.5, Applied Biosystems, USA) and the UCSC database. PCR products were electrophoresed on a 96-capillary automated DNA sequencer (MegaBACE 1000, Amersham, Germany) and were analyzed with Genetic Profiler software (version 1.5, Amersham, Germany). Two-point LOD scores were calculated using MLINK from the LINKAGE package (version 5.1). Autosomal dominant inheritance, disease-gene frequency of 0.0001, and 95% penetrance were assumed. Haplotyping was constructed using Cyrillic (version 2.1).

## Results

### Evaluation of exome sequencing data

A strategy of whole exome sequencing by hybrid capture and NGS was employed. The raw sequencing data obtained from the proband (II:6) was 9.4 Gb. The average read length was 90 bp. The efficiency of the hybrid capture was 81.6%; 71,968,280 out of 88,234,362 reads were uniquely mapped to targeted exome regions, and 99.39% of the whole exome was covered by reads. The distribution of per-base sequencing depth in target regions approximated a Poisson distribution, which showed that the captured exome region was evenly sampled (Additional file 1: Figure S1). Mean depth per base within the target regions was 111.85-fold, and 97.7% of these regions were covered by four or more reads (94.5% by 10 or more reads) by paired-end sequencing.

### Combinational analysis of exome and linkage identified the causative gene

A total of 1868 genetic variations, including non-synonymous mutations, splice site variations, and indels, were identified from the proband (Table 1). Because numerous mutations were detected, we combined whole exome sequencing and linkage analysis to sift through the potential causative mutations. As described previously by Gao et al. [11], five loci with positive but non-significant LOD scores (>1) were identified by linkage analysis (Additional file 1: Table S2).

**Table 1:** Variations identified by whole exome sequencing

**Mutation type**	**Number**
**SNP analysis**	
Missense	1363
Nonsense	34
Splice site	206
Readthrough	5
**Indel analysis**	
indel	37
Splice site	50
Frameshift	41
5-UTR	87
3-UTR	41
Promoter	4
**Total**	**1868**

**Figure 2:**
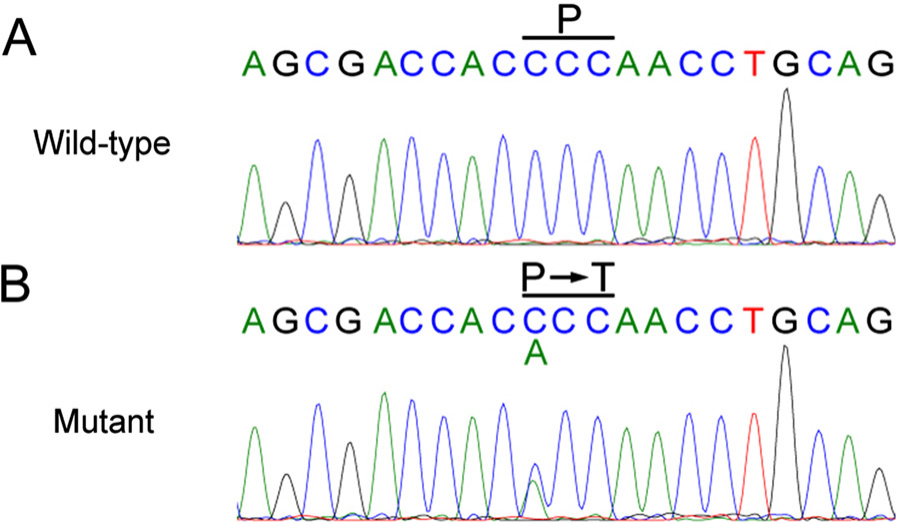
**Sequence and pedigree analysis of the C to A transversion in exon 2 of *CRYGD*. (A)** Sequence of the wild-type *CRYGD* alleles in the unaffected family members. **(B)** Heterozygous C to A mutation of *CRYGD* exon 2, resulting in a substitution from proline (P) to threonine (T), was detected in affected patients. A single transversion was observed as a C/A double peak.

In these five positive loci, we identified 11 mutations in nine genes by sequencing, including 10 SNPs and one short indel (Table 2). A mutation of *CRYGD*, a known gene causing congenital cataract, was included in these mutations. This mutation at nucleotide position c.70 in exon 2 of *CRYGD* (Figure 2) results in a threonine substitution for proline at amino acid residue 24 (P24T). The mutation was present in 10 patients but absent in 12 unaffected members of the studied family and in 100 control chromosomes from unaffected individuals of matched geographical ancestry. The remaining 10 mutations were present in both patients and healthy relatives and showed no co-segregation with the disease. We also screened 35 known cataract genes (Additional file 1: Table S1) and identified five mutations in *CRYGD*, *TMEM144*, *VIM*, *JAM3*, and *BFSP1* (Additional file 1: Table S3). These mutations also did not co-segregate with the disease, except for *CRYGD*.

**Table 2:** Variations identified in five candidate loci with LOD scores of 1-3

**Chromosome**	Position*	**Gene**	**Mutation type**	**Mutation**
chr2	198351787	*HSPD1*	SNP	G>A
chr2	198351850	*HSPD1*	SNP	G>A
chr2	198363406	*HSPD1*	SNP	G>A
chr2	202173902	*ALS2CR12*	SNP	C>T
chr2	208989018	*CRYGD*	SNP	C>A
chr2	210557406	*MAP2*	SNP	C>T
chr3	128859211	*ISY1*	SNP	C>A
chr3	129302495	*PLXND1*	SNP	C>T
chr3	133331276	*TOPBP1*	SNP	G>A
chr3	142031581	*XRN1*	SNP	G>A
chr3	127294786-127294788	*TPRA1*	Indel	-3GAA

*Coordinates based on the genome assembly hg19.

### Linkage analysis with newly sampled family members and haplotyping

Considering the new findings by exome sequencing, three additional individuals were newly recruited from the same pedigree, and the linkage analysis was performed once again. The maximum LOD score was obtained at D2S2237 (*Z*=3.53, *θ*=0.0; Table 3). Recombination events in several individuals defined the proximal and distal borders of a significant cataract-associated locus within the region between D2S309 and D2S2178 on chromosome 2q33-34 (Figure 1). These results also suggest that *CRYGD* is the causative gene for the coralliform cataract observed in this family.

**Table 3:** Two-point LOD score on chromosome 2q33-34 from the linkage analysis using all 22 family members

**Marker**	**Mb**	**LOD Score at*θ*=**					***Z***_**max**_	***θ***_**max**_
		**0**	**0.1**	**0.2**	**0.3**	**0.4**		
D2S364	183	-3.09	1.07	1.16	0.94	0.53	1.16	0.2
D2S117*^,#^	195.6	-4.25	0.39	0.37	0.23	0.09	0.39	0.1
D2S2318*	198.7	0.71	0.76	0.64	0.46	0.24	0.76	0.1
D2S309*	201.9	-3.96	1.23	1.08	0.77	0.39	1.23	0.1
D2S2237*	205.6	3.53	2.94	2.28	1.54	0.73	3.53	0.0
D2S325*^,#^	208.2	2.15	1.98	1.61	1.13	0.59	2.15	0.0
D2S2178*	209.8	-3.4	1.67	1.39	0.95	0.46	1.67	0.1
D2S1385*	210.4	-3.55	0.94	0.85	0.6	0.3	0.94	0.1
D2S2382^#^	217	-5.06	0.44	0.58	0.43	0.14	0.58	0.2
D2S2250	219.7	-9.82	-1.04	-0.26	0.01	0.06	0.06	0.4
D2S126^#^	221	-3.12	0.59	0.76	0.64	0.37	0.76	0.2
D2S206^#^	233.7	-5.77	-1.38	-0.71	-0.35	-0.14	-0.14	0.4

*Markers used for haplotyping.

^#^Markers used for initial gene scan to exclude the known loci.

## Discussion

We identified a C to A transversion at nucleotide position c.70 in exon 2 of *CRYGD* as the mutation responsible for congenital cataract in this family. CRYGD is an important structural protein essential for human lens transparency [15]. Based on the crystal structure of human CRYGD, the P24T mutation affects the N-terminal domain within the first Greek-key motif, causing the protein to have a slightly increased beta-sheet content, which may be attributed to the extension of an edge beta-strand due to the substitution of Pro24 with a residue capable of forming hydrogen bonds. The small increase in the fraction of beta-sheet content in the P24T mutant protein may contribute to the physical basis for precipitation of the protein [16,17]. The P24T mutation in the *CRYGD* gene has been found in several pedigrees with various cataract phenotypes, including cerulean, coralliform, and fasciculiform [18-20], lending support to the conclusion that the P24T mutation in *CRYGD* identified in this study is the cause of congenital cataract in the affected family.

In order to contribute to the technological progress of mapping genetic causes of human disease, whole exome sequencing should be fast, comprehensive, and economical to identify protein-coding mutations, including missense, non-sense, splice site, and small deletion or insertion mutations. However, an individual typically varies from the reference genome at over 10,000 potential mutations [21]. In this study, a total of 1868 genetic variations were identified by whole exome sequencing. Due to this high number, sifting through the hundreds of gene variations to identify the causal mutation would be a difficult task. Therefore, we combined whole exome sequencing with linkage analysis to identify the causative mutation in five loci with positive but non-significant LOD scores. The majority (99.4%) of mutations were excluded, and only 11 candidate mutations in these linkage loci were identified. Finally, we identified *CRYGD* as the gene responsible for the cataract phenotype in these 11 mutations. Our observations show that the linkage-exome combinational analysis is an efficient strategy for identification of pathogenic mutations by remarkably reducing the pool of candidate genes.

For further confirmation, we recruited another three members from the same family and selected an additional seven microsatellite markers for fine linkage analysis. Significant linkage was easy to establish with these three newly recruited members. The maximum LOD score (Z=3.53, *θ*=0.0) was obtained for marker D2S2237 near the *CRYGD* gene (Table 3). These results further suggested that *CRYGD* was responsible for the coralliform cataract in this family. However, recruiting new family members is not always possible. Therefore, using linkage analysis in a small nuclear family followed by exome sequencing of a single patient to identify the causative gene is a feasible method. According to the present observations, whole exome sequencing is promising for the analysis of monogenic diseases and allows identification of pathogenic mutations when the linkage score is not significant or the candidate regions are too large to be investigated.

## Conclusions

In conclusion, we identified the missense P24T mutation in *CRYGD*, which was responsible for the coralliform cataract afflicting a four-generation Chinese family. Notably, our study indicated that the linkage analysis-whole exome sequencing approach is a powerful tool for finding pathogenic genes of Mendelian inheritance and provides important guidance for developing an analytical framework in the near future.

## Competing interests

The authors declare that they have no competing interest.

## Authors’ contributions

SF obtained the funding and designed the research. XJ carried out the laboratory work, analyzed most of the data, and drafted the manuscript. FZ participated in the design of the research and revision of the manuscript. JB and YY carried out sample collection and helped prepare the laboratory work. LG revised the manuscript and did the linkage analysis. WS supervised the research personnel. XZ and HS participated in the sequence alignment and revised the manuscript. RG and LX analyzed and interpreted the data. ZY and DS carried out the molecular biology experiments. All authors have read and approved the final manuscript.

## Pre-publication history

The pre-publication history for this paper can be accessed here:

http://www.biomedcentral.com/1471-2350/14/107/prepub

## Supplementary Material

Additional file 1: Table S1 Known genes involved in congenital cataract. **Table S2.** Two-point LOD score (> 1) for the microsatellite markers in our previous study on 19 family members. **Table S3.** Variations identified in 35 known cataract genes. **Figure S1.** The read-depth distribution of exome sequencing assay. X-axis shows sequencing depth, while Y-axis indicates the percentage of total target regions under a given sequencing depth [22-56]. Click here for file
