# Supplementary material for: Combinational analysis of linkage and exome sequencing identifies the causative mutation in a Chinese family with congenital cataract
Source: BMC Med Genet. 2013 Oct 8;14:107. doi: 10.1186/1471-2350-14-107 (PMC3851584; doi:10.1186/1471-2350-14-107)
Supplement: Additional file 1: Table S1 — Known genes involved in congenital cataract. Table S2. Two-point LOD score (> 1) for the microsatellite markers in our previous study on 19 family members. Table S3. Variations identified in 35 known cataract genes. Figure S1. The read-depth distribution of exome sequencing assay. X-axis shows sequencing depth, while Y-axis indicates the percentage of total target regions under a given sequencing depth [22-56]. [file 1471-2350-14-107-S1.doc]

# Additional file 1

**Table S1. Known genes involved in congenital cataract**

| **Locus** | **Gene** | **Inheritance*** | **References** |
| --- | --- | --- | --- |
| 1p36 | *EPHA2* | AD |  |
| 1p32 | *FOXE3* | AD |  |
| 1q21.1 | *GJA8* | AD / AR |  |
| 2p25.3 | *PXDN* | AR |  |
| 2q33-35 | *CRYGA* | AD |  |
| 2q33-35 | *CRYGC* | AD |  |
| 2q33-35 | *CRYGD* | AD |  |
| 3p21.31 | *FYCO1* | AR |  |
| 3q21-25 | *BFSP2* | AD |  |
| 3q27 | *CRYGS* | AD |  |
| 5q31 | *SIL1* | AD / AR |  |
| 6p24 | *GCNT2* | AR |  |
| 8q13.3 | *EYA1* | AD |  |
| 10p13 | *VIM* | AD |  |
| 10q23.31 | *SLC16A12* | AD |  |
| 10q25 | *PITX3* | AD |  |
| 11q22.3-23.1 | *CRYAB* | AD / AR |  |
| 11q25 | *JAM3* | AR |  |
| 11p13 | *PAX6* | AD |  |
| 12q13 | *MIP* | AD |  |
| 13q11 | *GJA3* | AD |  |
| 16q13.2 | *TMEM114* | AD |  |
| 16q22-23 | *MAF* | AD |  |
| 16q21-22.1 | *HSF4* | AD / AR |  |
| 17q11.1-12 | *CRYBA1* | AD |  |
| 17q11.1-12 | *CRYBA3* | AD |  |
| 19q13.4 | *LIM2* | AR |  |
| 19q13.3-4 | *FTL* | AD |  |
| 20p12.1 | *BFSP1* | AR |  |
| 20q11.21 | *CHMP4B* | AD |  |
| 21q22.3 | *CRYAA* | AD / AR |  |
| 22q11.2-13.1 | *CRYBA4* | AD |  |
| 22q11.2-12.1 | *CRYBB1* | AD / AR |  |
| 22q11.2-12.1 | *CRYBB2* | AD |  |
| 22q11.2-12.2 | *CRYBB3* | AR |  |

*AD, autosomal dominant; AR, autosomal recessive.

**Table S**2. Two-point LOD score (> 1) for the microsatellite markers in our previous study on 19 family members

| LOD Score at *θ* = | | | | | | | | |
| --- | --- | --- | --- | --- | --- | --- | --- | --- |
| Marker | Mb | 0.0 | 0.1 | 0.2 | 0.3 | 0.4 | *Z*_max_ | *θ*_max_ |
| D2S319 | 3.4 | 2.09 | 1.68 | 1.23 | 0.75 | 0.29 | 2.09 | 0.0 |
| D2S2211 | 7.5 | 2.69 | 2.19 | 1.63 | 1.01 | 0.36 | 2.69 | 0.0 |
| D2S162 | 8.9 | 1.13 | 0.90 | 0.65 | 0.39 | 0.15 | 1.13 | 0.0 |
| D2S325 | 208.2 | 1.86 | 1.73 | 1.41 | 1.0 | 0.52 | 1.57 | 0.0 |
| D3S1292 | 131.6 | -2.58 | 0.90 | 1.04 | 0.87 | 0.52 | 1.04 | 0.2 |

**Table S**3. Variations identified in 35 known cataract genes

| Chromosome | Position (bp) | Gene | Mutation type | Mutation |
| --- | --- | --- | --- | --- |
| chr2 | 208,989,018 | *CRYGD* | SNP | C>A |
| chr4 | 159,140,503 | *TMEM144* | SNP | C>T |
| chr10 | 17,271,563 | *VIM* | SNP | A>C |
| chr11 | 134,014,164 | *JAM3* | SNP | G>A |
| chr20 | 17,475,093 | *BFSP1* | SNP | T>C |


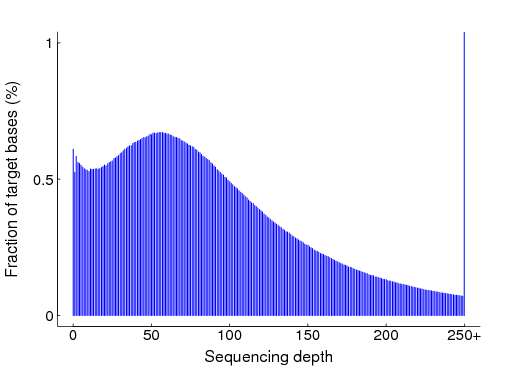


**Figure S1. The read-depth distribution of exome sequencing assay.** X-axis shows sequencing depth, while Y-axis indicates the percentage of total target regions under a given sequencing depth.
